# Polymerase Chain Reaction molecular diagnostic technology for monitoring chronic osteomyelitis

**DOI:** 10.1186/s40634-014-0009-6

**Published:** 2014-08-15

**Authors:** Brian D Mariani, Daniel S Martin, Antonia F Chen, Haruyo Yagi, Sheldon S Lin, Rocky S Tuan

**Affiliations:** Department of Orthopaedic Surgery, Thomas Jefferson University, Philadelphia, PA USA; Center for Cellular and Molecular Engineering, Department of Orthopaedic Surgery, University of Pittsburgh School of Medicine, 450 Technology Drive, Pittsburgh, PA 15219 USA; Molecular Infectious Disease Laboratory, Genetics & IVF Institute, 3015 Williams Drive, Fairfax, VA 22031 USA; Prime Health Network, 9 N Brookside Road, Springfield, PA 19064 USA; Department of Orthopaedic Surgery, Rutgers-New Jersey Medical School, 90 Bergen Street Room 1200, Newark, NJ 07101 USA

**Keywords:** Polymerase chain reaction, Osteomyelitis, Molecular diagnosis, Bacterial infection, *Staphylococcus aureus*

## Abstract

**Background:**

Osteomyelitis is a devastating condition whose treatment relies on the detection of bacteria. The current standard of microbiology culture may not be adequate. Molecular biology based diagnostic procedures for detecting bacteria in orthopaedic infections was previously established, but has not been applied to the setting of chronic osteomyelitis. We aim to determine the applicability of molecular diagnostic procedures for monitoring chronic osteomyelitis, and to evaluate if these procedures are superior to standard culture methods of osteomyelitis detection.

**Methods:**

A rabbit experimental model of chronic osteomyelitis was used; infection was induced in the proximal, medial aspect of the tibia with *Staphylococcus aureus* at titers ranging from 1 × 10^2^ to 1 × 10^6^ colony forming units. At 28 days post-infection, animals were sacrificed, and the tibias were examined radiographically, harvested, and assayed for the presence of bacteria. Two bacterial detection methods were used: (1) standard microbiological culturing, and (2) polymerase chain reaction (PCR) based diagnostic method to detect bacterial genomic DNA.

**Results:**

The molecular diagnostic method was highly sensitive and accurate, and detected low titer infections that were undetected by radiographic and microbiological methods. By using two sets of PCR primers, one for a universal bacterial gene (16S rRNA) and one for a species-specific gene (*nuc*), the molecular protocol allowed both the detection and speciation of the bacterial infection.

**Conclusions:**

The use of the PCR-based method was effective for high-sensitivity detection and identification of bacteria associated with chronic osteomyelitis in a rabbit model. Our findings illustrate the applicability of PCR for monitoring chronic osteomyelitis, which may be useful for improved detection of osteomyelitis organisms in humans.

## Background

Chronic osteomyelitis is a significant source of patient morbidity in both pediatric and adult populations [1,2]. The successful management of the disease is critically dependent on the timely and accurate diagnosis of infection as the etiology of the symptoms, and the ability to monitor effective antimicrobial or other curative therapies. Early detection of bacterial infiltration in bone, when bacterial titers are low and when standard diagnostic tests may be equivocal early in the infection cycle [3], could allow for more effective treatments to be instituted sooner to achieve bacterial infection eradication in osteomyelitis. However, standard culture methods may not be very effective for detecting orthopaedic infections [4].

Previous studies in our laboratory and others have demonstrated the superior sensitivity and accuracy of molecular biological technology, based on the DNA amplification technique of polymerase chain reaction (PCR); [5,6]) and for the detection of infection in symptomatic arthroplasty patients (Mariani et al. 1996b; [7–12]). Our recent studies and those of others have further refined molecular techniques to access bacterial viability in simulated and clinical infections [13–16]. Many other clinical fields are also developing protocols utilizing nucleic acid based methods for the detection of pathogens in the clinical setting [17–20]. For orthopaedics, enhanced diagnostics for infectious diseases could play a significant role in treating infections, especially in cases where the diagnosis of infection is not straightforward, such as fracture nonunions [21], painful prosthetic joints (Mariani et al. 1996b), loose prosthetic joints [22], certain arthritic conditions [23], and osteomyelitis subsequent to bone trauma [24] or as a result of secondary infection from hematogenous sources.

A number of animal models have been developed to investigate the pathogenesis of osteomyelitis and the efficacy of various antibiotic treatment regimens [25,26]. Previously, our laboratory has used the rabbit experimental osteomyelitis protocol developed by Norden [27] to test the effectiveness of a biodegradable carrier for the local delivery of antibiotics to the site of infection in a fracture stabilization model [28–30]. In these studies, chronic infection was routinely established in 14 to 28 days using an inoculum of 1 × 10^6^ colony forming units (CFU) of *Staphylococcus aureus* (strain 23923, American Type Culture Collection, Manassas, VA).

In the present study, we sought to use the experimental osteomyelitis model in rabbits [27] to test the effectiveness of the PCR-based molecular diagnostic assay, particularly in detecting low level of infection in the early stages of disease progression. In this study, we addressed the following questions: (1) Is the molecular diagnostic procedure applicable for monitoring chronic osteomyelitis?; and (2) Does molecular diagnostic perform better than culture methods for osteomyelitis detection?

## Methods

### Experimental chronic osteomyelitis

Experimental osteomyelitis was induced in rabbits using *Staphylococcus aureus* (strain 23923, American Type Culture Collection, Rockville, MD) according to the protocol of Norden [27].

#### Bacterial growth

Bacteria were grown overnight under aerobic conditions in tryptic soy broth at 37°C, concentrated by centrifugation, washed, and resuspended in a one sixth volume of sterile physiological saline. Bacterial concentration was estimated spectrophotometrically on the basis of optical density at 600 nm (1 OD600 unit = 1 × 10^9^ cells/mL). Additionally, CFU titers of suspension were verified by plating aliquots from a ten-fold dilution series on tryptic soy agar plates in duplicate and counting colonies after overnight growth at 37°C. Bacterial suspensions of 1 × 10^6^, 1 × 10^4^, 1 × 10^3^, and 1 × 10^2^ CFU/0.1 ml were prepared by serial dilution, and 0.1 ml of each concentration was used for injection into the medullary cavity of rabbit tibias prepared as described below.

#### Animal procedure

Approval was obtained from the Institutional Animal Care and Use Committee (Thomas Jefferson University IACUC) (Protocol 234 J) prior to performing this study. New Zealand White rabbits weighing 2–3 kg were anesthetized, their left tibias shaved and cleaned with alcohol, and the bone exposed by a small incision. An 18 gauge needle was inserted through the medial aspect of the tibia 1–2 cm distal from the proximal metaphysis to deliver, in order, a 0.1 ml volume of 5% sodium morrhurate as a sclerosing agent, a 0.1 ml volume of bacterial suspension of appropriate concentration (see above), and a 0.1 ml volume of sterile saline to facilitate the entry of the mixture into the medullary cavity, after which the wound was closed. Animals were administered analgesics postoperatively, and monitored during the recovery period until their mobility returned. Those animals displaying signs of discomfort were given analgesics as necessary. Osteomyelitis was usually established between 14 and 28 days, depending on the inoculum titer [31–33]. Animals were sacrificed at 28 days for all inoculum titers.

Experimental osteomyelitis was induced in 15 rabbits using *Staphylococcus aureus* at 4 different inoculum titers: 1 × 10^6^ (animals #1,2,3,4), 1 × 10^4^ (#5,6,7,10,11,12,13), 1 × 10^3^ (#14), and 1 × 10^2^ (#15,16,17) CFU. Three additional animals were used as negative controls: two animals were mock infected with the sclerosing agent and saline alone (#8,18), and 1 animal was not operated on (#9). Animals were sacrificed at 28 days, and either one (Group 1: Animals #1-9) or four (Group 2: Animals #10-18) biopsies were obtained from each animal as described below.

#### Analysis of infection

Infection was analyzed radiographically, as well as using microbiological and molecular assays. Biopsy of the bone at the site of infection was performed in one of two ways. For the first group of 9 rabbits, the operated tibia was surgically retrieved, dissected free of soft tissue, alcohol cleaned, and a biopsy of bone and bone marrow was recovered from the osteotomy site (where infection was induced) using a curette. For the remaining animals (Group 2, 9 animals), four biopsy methods were used. In addition to the first retrieval of bone at the osteotomy site as described above, a second bone and bone marrow biopsy was recovered from the immediate, lateral metaphysis in a similar manner, to test for the migration of infection to this region. In addition, in this second group of animals, prior to the biopsy procedure described above, aspirate samples were taken from both regions (osteotomy and metaphysis) using a 18 gauge needle to retrieve a specimen containing both cortical bone and bone marrow. All retrieved materials were weighed and divided into two aliquots and subjected to microbiological culture and DNA extraction for molecular analysis in parallel. Radiographs were taken of all operated limbs in situ prior to specimen retrieval.

### Microbiological culture

All biopsy and needle retrieval specimens were homogenized and mixed in 1.0 ml of sterile physiological saline, diluted serially in saline in triplicate, and then plated on tryptic soy agar for colony counts after incubation at 37°C. Bacterial growth was not assessed quantitatively, since there was quantitative variability in the recovered specimen.

### Molecular diagnosis

DNA extraction was carried out using a previously established protocol [6] DNA was extracted from the portion of the specimen not used for microbiological culture as follows. The specimen was finely minced with sterile scissors, added to 0.2 ml extraction buffer containing 100 mM Tris–HCl, pH 8.0, 2 mM EDTA, pH 8.0, 50 mM KCl, 0.5% (vol/vol) Tween 20, and 0.5% (vol/vol) NP-40, mixed vigorously by vortexing, and heated to 95°C for 10 minutes. After cooling to room temperature, a mixed bed ion exchange resin (BioRad AG501-X8, Hercules, CA) was added to a final concentration of 20% (wt/vol), mixed by vortexing, cleared by centrifugation for 10 minutes at 10,000 × g, and the supernatant containing the extracted nucleic acid was transferred to a new tube and stored until PCR testing.

#### Polymerase Chain Reaction (PCR)

As described previously [6], PCR was performed on all specimens using 5 μl of extract in a 50 μl reaction volume using 0.25 units of AmpliTaq LD DNA polymerase (Perkin Elmer, Norwalk, CT) per reaction using the manufacturer’s buffer system according to the manufacture’s specifications. The nucleic acid amplification reactions were performed in a thermocycler (MJ Research DNA Engine Thermocycler, Watertown, MA) using the following cycling profile, following a pre-cycle incubation at 94°C for 4 min: denaturation at 94°C for 1 min, annealing at 55°C for 1 min, and primer extension at 72°C for 2 min, for 30 cycles, using 50 pmol of each amplification primer per reaction. Two different primer sets were used for bacterial detection in this study (Integrated DNA Technologies, Coralville, IA). The sequences for the forward and reverse primers for the first set, complementary to the conserved, multicopy 16S ribosomal ribonucleic acid (rRNA) gene, were 5′-CGGCAGGCCTAACACATGCAAGTCG-3′, and 5′-GGTTGCGGCCGTACTCCCCAGG-3′, respectively. The sequences for the forward and reverse primers of the second set, complementary to the *Staphylococcus aureus* specific heat stable nuclease gene *(nuc)*, were 5′-5′ GCG ATT GAT GGT GAT ACG GTT-3′ and 5′ AGC CAA GCC TTG ACG AAC TAA AGC-3′, respectively. PCR products were analyzed by agarose gel electrophoresis and ethidium bromide staining, followed by membrane blotting and hybridization analysis using either the [^32^P]-labelled 16S rRNA or *nuc* gene fragment as hybrization probe [34,35].

### Comparison of quantitative real-time PCR to PCR

A quantitative evaluation of the DNA-based PCR procedure used here was also made by comparison with our recent protocols using real time quantitative PCR (qPCR) [13,14].

#### Reagents

DNeasy Blood & Tissue kit was purchased from QIAGEN (Gaithersburg, MD). SYBR Green PCR Master Mix was purchased from Invitrogen (Grand Island, NY). PrimeSTAR HS was purchased from Clontech Laboratories (Mountain View, CA). 16S rRNA primers were obtained from Integrated DNA Technologies (forward: 5′-ATTAGATACCCTGGTAGTCCACGCC-3′; reverse: 5′-CGTCATCCCCACCTTCCTCC-3′; 387 base-pair amplification product).

#### Bacterial culture

*Escherichia coli* HB101 (ATCC 33694) were grown in LB medium at 37°C and bacterial concentration (cells/mL) was determined based on OD 600 (1 OD600 unit = 1 × 10^9^ cells/mL). Final bacterium concentration was 1.98 × 10^9^ cells/mL.

#### DNA isolation

Genomic DNA was extracted from a 5 mL aliquot of *E. coli* HB101 culture using the DNeasy Blood & Tissue kit (QIAGEN). The DNA content was measured (based on OD 260 nm) and the DNA/cell value was subsequently used to estimate cell number.

#### PCR

PCR was performed using the PrimeSTAR HS kit (Clontech Laboratories). Ten-fold serial dilutions of template DNA were used to generate the standard curve. Fifty μL of PCR reaction mixture consisted of 10 μL of genomic DNA (0, 10^−4^, 10^−3^, 10^−2^, 10^−1^, 1, 10^1^, 10^2^ ng), 4 μL of 2.5 mM dNTP Mixture, 25 μL of primeSTAR buffer, 2.5 μL of 5 μM 16S rRNA primers (mixture of forward and reverse primers), 0.5 μL of 2.5 units/μL primeSTAR HS DNA polymerase, and 8 μL of distilled water. The control sample did not contain genomic DNA. PCR was performed with the Applied Biosystems Veriti 96 well Thermal Cycler (Life Technologies, Grand Island, NY) as follows: 95°C for 4 min.; 35 cycles of 95°C for 20 s, 58°C for 30 s, 72°C for 45 s; 72°C for 5 min; 4°C for infinity. After amplification, a 5 μL aliquot of each sample was directly subjected to gel electrophoresis in 2% agarose gel in the presence of ethidium bromide. Image J (NIH) was used to quantify the levels of each sample. All reactions were performed in triplicates using the same genomic DNA stock.

#### Quantitative Real-time PCR

qPCR was performed with SYBR Green Master Mix using ABI StepOne Real-Time PCR Systems (Life Technologies, Grand Island, NY). Five-fold serial dilutions of template DNA were used to generate the standard curve. 20 μL of qPCR reaction mixture consisted of 5 μL of genomic DNA (0, 1.28 × 10^−4^, 6.4 × 10^−4^, 3.2 × 10^−3^, 1.6 × 10^−2^, 8 × 10^−2^, 4 × 10^−1^, 2, 10, 50 ng), and 1 μL of 5 μM 16S rRNA primers (mixture of forward and reverse primers), 10 μL of SYBR Green Master Mix, and 4 μL of distilled water. The control sample did not contain genomic DNA. The cycling conditions were 95°C for 10 min, followed by 45 cycles of 95°C for 10 s ad 60°C for 30 s. For all samples, the cycle number at which the fluorescence values became logarithmic (Ct) was determined. The ΔCt value (sample Ct-control Ct) was calculated for each sample as the difference between the sample Ct and control Ct. All reactions were performed in quadruplicates using the same genomic DNA stock.

## Results

### Microbiological culture analysis

In Group 1 animals that received a single biopsy, 6 out of the 7 (86%) experimental animals gave positive microbiological cultures (Table 1). For the remaining experimental animals (Group 2) that were tested for infection using the four biopsy method, 4 of the 8 rabbits produced at least 1 microbiological culture test that was positive for bacterial growth, but none tested positive for all four biopsy methods (Table 2). Of the 4 animals that received 1 × 10^4^ CFU inoculations in Group 2, 3 tested positive in one or more biopsies and 1 tested negative for bacterial culture growth. The 1 animal that received a 1 × 10^3^ inoculation was negative. Finally, in 1 of the 3 animals receiving 1 × 10^2^ CFU inoculations, bacterial growth was detected with 1 biopsy method in 1 animal, but no growth was detected in the other 2 animals.

**Table 1:** **Rabbit Osteomyelitis (Group 1): comparison of infection detection methods**

		**Infection detection**		
**Animal no.**	**Inoculum (CFU)**	**Microbiological culture**	**Radiography**	PCR test*
1	1 × 10^6^	+	+	+
2	1 × 10^6^	+	+	+
3	1 × 10^6^	+	+	+
4	1 × 10^6^	+	+	+
5	1 × 10^4^	+	+	+
6	1 × 10^4^	-	+	+
7	1 × 10^4^	+	+	+**
8	Sclerosing agent and saline only	-	-	-
9	Unoperated control	-	-	-

*PCR done using 16S rRNA gene primers.

**Positive PCR result obtained after two rounds of extraction of sample with Mixed Bed Ion Exchange Resin (see text for details).

**Table 2:** **Rabbit Osteomyelitis (Group 2): comparison of infection detection methods**

			Microbiological culture and PCR*							
			**Metaphysis biopsy**				**Osteotomy biopsy**			
			**Needle**		**Curette**		**Needle**		**Curette**	
**Animal no.**	**Titer (CFU)**	**Radiograph**	**Culture**	**PCR**	**Culture**	**PCR**	**Culture**	**PCR**	**Culture**	**PCR**
10	1 × 10^4^	+	-	+	+	-	+	+	+	+
11	1 × 10^4^	+	-	+	+	+	-	+	-	+
12	1 × 10^4^	+	+	+	-	-	+	+	-	-
13	1 × 10^4^	+	-	-	-	+	-	+	-	-
14	1 × 10^3^	-	-	+	-	-	-	+	-	+
15	1 × 10^2^	-	-	+	-	-	-	-	-	+
16	1 × 10^2^	-	-	-	-	+	-	+	-	-
17	1 × 10^2^	-	-	-	+	+	-	-	-	-
18	Sclerosing agent/saline only	-	-	-	-	-	-	-	-	-

*PCR done using *Staphylococcus aureus nuc* gene primers.

No bacterial growth was recovered from the 3 negative control animals used in this study. In total, of the 15 inoculated experimental animals tested, 10 (67%) gave at least one biopsy that was positive for bacterial infection. However, in the 39 total microbiological culture tests performed on the 15 animals, taking into account the animals with multiple biopsies, only 13 tests were positive, yielding a 33% sensitivity, assuming infection was established in all animals at all sites. [Note: Verification that the bacteria recovered from the culture assays was *Staphylococcus aureus* was achieved by PCR amplification of DNA extracted from individual colonies from a representative number of culture plates using the *Staphylococcus aureus-*specific *nuc* gene primers, carried out as described below. Amplification of the expected gene fragment in all cases indicated infection was not due to a secondary infection by commensal, coagulase-, or nuclease-negative *Staphylococci* bacteria].

### Radiographic analysis

**Figure 1:**
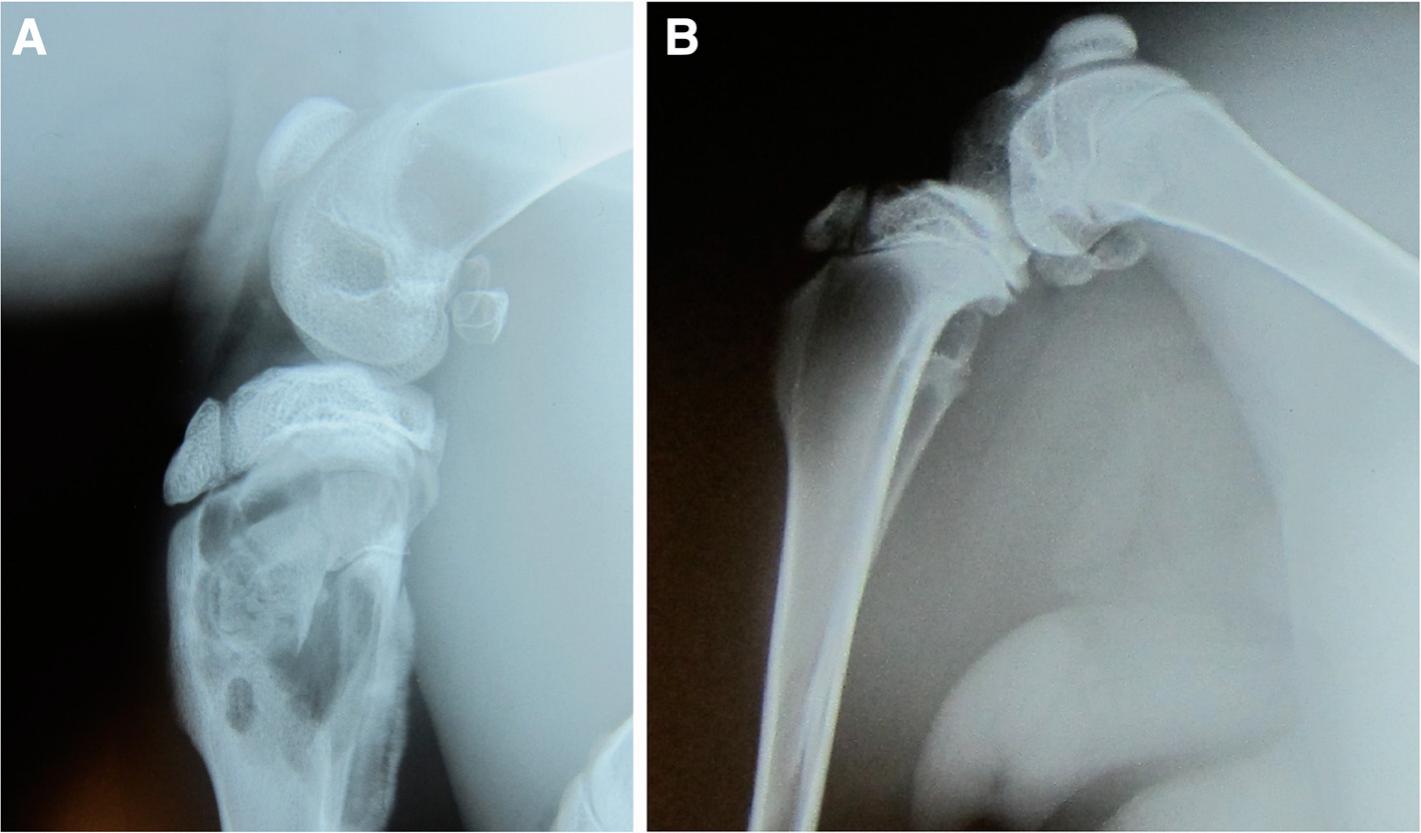
**Radiographic evaluation of osteomyelitis. (A)** A proximal rabbit tibia that had been inoculated with 1 × 10^6^ CFU of bacteria exhibits evidence of osteomyelitis, characterized by lucency and bony erosion. **(B)** A proximal rabbit tibia that had not been inoculated with bacteria demonstrates no evidence of osteomyelitis or bony compromise.

Examination of the radiographs taken from these animals showed distinct lucency and evidence of bone erosion only in the animals inoculated with 1 × 10^6^ CFU of bacteria (Animals #1-4, Figure 1A). All other animals failed to exhibit any overt signs of compromised bone quality, in a double-blind examination (Figure 1B).

### PCR-based molecular analysis

The remaining half of all biopsy materials recovered from inoculated and control animals was processed for total nucleic acid extraction using a rapid protocol previously described for synovial fluid specimens from symptomatic arthroplasty patients [6]. The presence of bacterial infection was documented by PCR amplification of DNA recovered from biopsies using primers specific for two different bacterial genes: (1) the bacteria-specific, conserved, multi-copy 16S rRNA gene; or (2) the *Staphylococcus aureus-*specific nuclease *(nuc)* gene. For the single biopsy group (Group 1), comprising all animals inoculated with 1 × 10^6^ CFU, and 3 of the animals inoculated with 1 × 10^4^ CFU of bacteria, the 16S rRNA PCR primers were used for amplification of bacterial DNA. After agarose gel electrophoresis and blot hybridization analysis using cloned 16S rRNA gene fragment as hybridization probe, 6 of the 7 infected animals were positive for infection based on PCR. The mock inoculated and unoperated negative control animals were negative using the PCR test.

A positive control PCR reaction was run on all specimens, which entailed the addition of an internal control DNA template containing the same 16S primer sequences to each amplification reaction mixture to verify the ability of all extracts to support DNA amplification. In all cases, except for rabbit #7, positive control product was detected from the extracts. This result indicates that the extract derived from rabbit #7 contained substances inhibitory to the Taq DNA polymerase enzyme, and additional processing was required. Since this specimen contained a large proportion of whole blood, the extract was retreated with the ion-exchange mixed bed resin, and a second round of amplification was performed and bacterial derived PCR product was subsequently detected. This result indicated that infection was evident and detectable by PCR after additional processing, and increased the PCR detection sensitivity to 100% (7/7) for this set of animals.

For Group 2, the animals tested for infection using the four biopsy method, PCR analysis was performed with the *nuc* gene amplification primers to directly establish *Staphylococcus aureus* as the infectious agent. In this group, PCR product was detected from all infected animals, for either some, or all the biopsy methods. There was a general correlation between the signal intensity and number of positive tests per animal, and the concentration of the initial bacterial inoculation. In 3 of 4 animals receiving 1 × 10^4^ CFU, and the 1 animal receiving 1 × 10^3^ CFU, 3 or more biopsy methods gave strong positive PCR signals for each animal. In the 3 animals receiving 1 × 10^2^ CFU inoculations, only 1 or 2 of the four biopsy methods used for each animal yielded PCR signal. In total, 25 of the 39 infection tests were positive as determined by PCR. The usefulness of the *Staphylococcus aureus-*specific nuclease *(nuc)* gene primers in the amplification reaction was two-fold: first, amplification and detection of bacterial DNA product indicated infection was indeed present, and secondly, it allowed the unequivocal determination of *Staphylococcus aureus* as the infectious agent.

**Figure 2:**
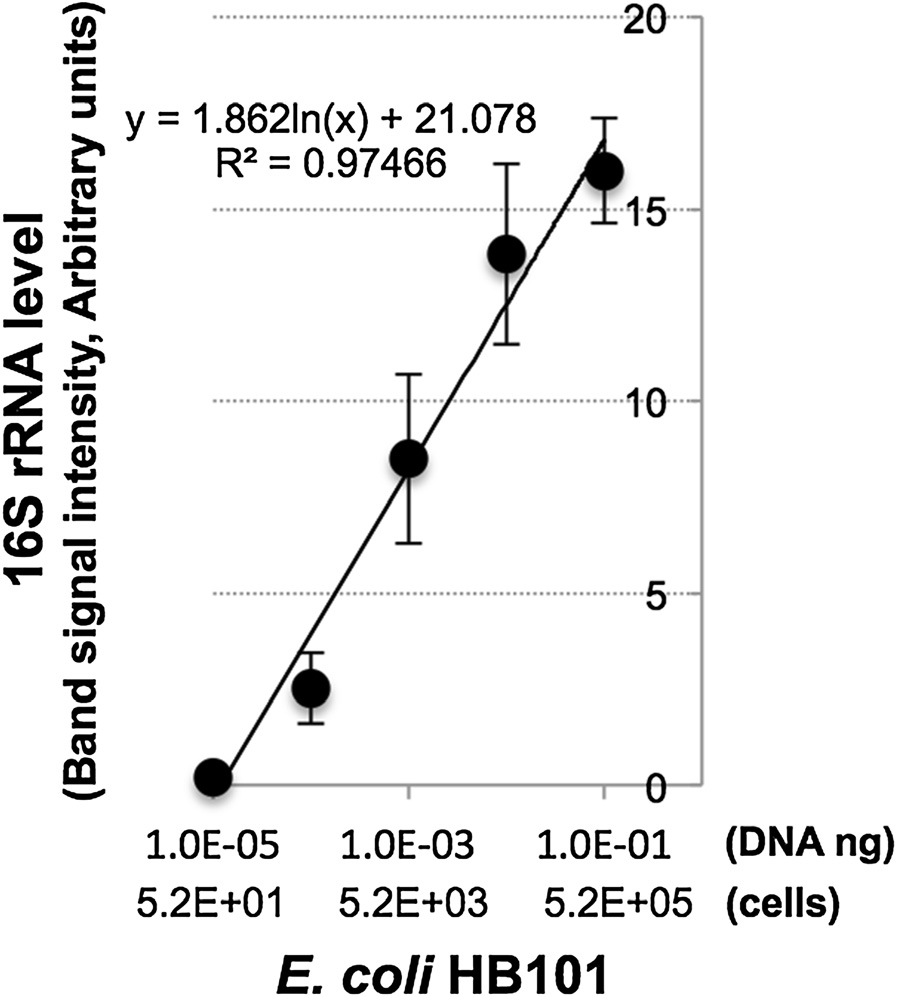
**PCR standard curve for 16S rRNA using diluted** ***Escherichia coli*** **HB101 genomic DNA as a template.** Linear regression fit performed on the standard curve in the concentration range from 10^−5^ to 10^−1^ ng DNA.

**Figure 3:**
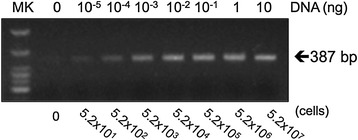
**Gel electrophoresis analysis of PCR product from 16S rRNA.** Ethidium bromide staining indicated sensitivity of detection to ~50 cells.

**Figure 4:**
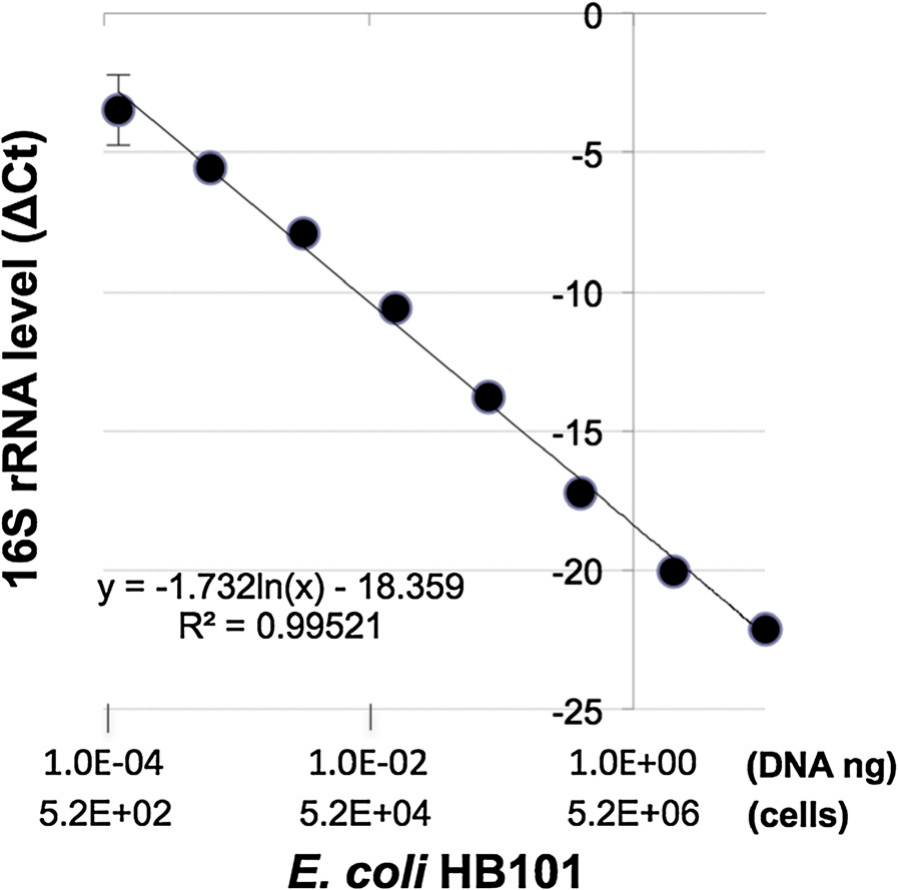
**qPCR quantitative analysis of 16S rRNA.** *E. coli* genomic DNA was used as a template. Linear regression fit performed on the standard curve in the concentration range from around 10^−4^ to 10 ng DNA.

The detection limits of PCR & qPCR for bacterial 16S rRNA were next assessed using *Escherichia coli* DNA samples extracted from a known bacterial titer. Traditional PCR method based on gel electrophoresis analysis gave linear standard curve in the concentration range from 10^−4^ to 10^−1^ ng DNA (equivalent to 5.2 × 10^2^ to 5.2 × 10^5^ cells) (Figures 2 and 3), but showed apparent saturation at higher amounts of DNA (i.e., 1 and 10 ng, data not shown). In comparison, qPCR method yielded linear standard curve in the concentration range from around 10^−4^ to 10 ng DNA (equivalent to 5.2 × 10^2^ to 5.2 × 10^7^ cells) (Figure 4). Our results therefore showed that qPCR readily produced quantitative results and could detect 100 times higher bacteria concentration without loss of sensitivity or linearity of detection compared to the conventional PCR method.

### Comparison of microbiological, radiographic and molecular analyses

For the animals in the 1 × 10^6^ CFU inoculation group, there was concordance between all three diagnostic methods. However, infection testing of the animals in the 1 × 10^4^ or lower CFU groups showed discordance between the three methodologies. Microbiological culture assays detected infection in only 5 of 7 animals receiving 1 × 10^4^ CFU inoculations. In the 4 animals of that group that were assayed using the multiple biopsy methods, only a subset of the biopsies for each animal gave bacterial growth. Radiographic examinations failed to provide any definitive diagnosis of infection. On the other hand, in these 1 × 10^4^ CFU inoculation animals, the PCR test was positive in all 7 animals, 3 of which were positive with the four biopsy method. In the 1 × 10^3^ CFU animal, and the 3 animals receiving 1 × 10^2^ CFU, a greater number of positive PCR tests were obtained compared to culturing. In these low titer infections, microbiological culture was positive in only 1 of 16 biopsies (rabbit #17), compared to 8 positive PCR results. In only one case (rabbit #10) was there a biopsy positive using microbiological culture, but negative by PCR, although all other biopsies of that animal were PCR positive. Overall, the molecular test displayed a substantially greater sensitivity compared to the microbiological test or radiographic examination, and the results were available within 24 hours, or less, after specimen collection.

## Discussion

Osteomyelitis continues to be a troublesome infection affecting pediatric patients and adults alike, eliciting fever, pain, inflammation, and bone degradation and loss, which sometimes can be fatal if not properly addressed with antimicrobial, or surgical intervention. Whereas acute osteomyelitis subsequent to bone trauma or a surface wound infection may be readily diagnosed, slowly progressing chronic osteomyelitis derived from hematogenous sources often presents a more difficult diagnosis [1,2]. Experimental osteomyelitis in animals [27,36], for example using the rabbit model, has been valuable for studying the establishment, progression and treatment of the chronic disease. Several different protocols have been employed for the induction of chronic osteomyelititis. We have successfully employed the rabbit model developed by Norden [27] that uses a sclerosing agent to promote the establishment and progression of infection. In our previous study [28], the rabbit model has been used to test the efficacy of a biodegradable antibiotic delivery system for the treatment of bone infection. The success of this model prompted us to use it to test the efficacy of the protocol we have established for infection detection using molecular biology methodology ([6,8]; Mariani et al. 1996b; [7,13,14]). Experimental osteomyelitis in this animal model allows the precise control of infectious dose, progression of disease, choice of infectious agent, and conditions that are amenable for the rigorous testing of new diagnostic strategies. Thus, the purposes of our study were to determine (1) the applicability of the molecular diagnostic procedure for monitoring chronic osteomyelitis, and (2) whether molecular diagnostic is superior to culture methods of osteomyelitis detection.

The molecular test demonstrated excellent sensitivity in two respects. First, all infected animals were determined to be positive by PCR, and secondly, infection documentation was unequivocal in all animals displaying low level disease. In these latter animals, standard microbiological and radiographic testing failed to establish either the presence of bacterial infiltration, or morphological evidence of pathological involvement of bone. It could be argued that these animals might not have had, or were not going to progress to, established chronic osteomyelitis, and potentially would have cleared the microorganisms immunologically, and that the PCR test was detecting nucleic acid from the original bacterial population in the inoculum, and not focal disease resulting from pathogenic proliferation in vivo. However, it is highly unlikely that any organisms from the inoculum would have remained intact in a healthy, immunoreactive animal for 4 weeks, without clearance by the immune system, to result in false positive PCR results. More plausible is that growth and expansion of the inoculum bacterial population did occur in these animals, and that even though classic osteomyelitis was not fully established based on standard radiographic evidence (Figure 1), underlying bacterial proliferation and colonization that would eventually manifest as chronic disease was indeed taking place, and was in fact detectable by the more sensitive molecular assay, but not by microbiological or radiographic analysis. These findings demonstrate that the molecular test, if used appropriately in clinical situations where osteomyelitis is suspected, or where a definite probability of its development exists because of the clinical history of the patient, could be a valuable diagnostic assay for detecting low bacterial loads in the early stages of infection, prior to fully developed disease, at a time when antimicrobial therapy would be the most effective.

Two different amplification primers strategies have been used in this study. With one set of animals, the “universal” 16S rRNA gene primers that target a conserved region of this multi-copy gene unique to bacteria were used for bacterial detection. These primers hybridize to the 16S rRNA gene of essentially all pathogenic bacteria and are useful for broad spectrum detection of infection in situations where the infectious agent cannot be easily cultured, or in infections that can be caused by one of many different species that all present with similar, indistinguishable symptoms. Although a predominant number of clinical osteomyelitis cases in humans are caused by *Staphylococcus aureus*, other species are routinely isolated, including *Streptococcus pyogenes* and other Group A Streptococci, *Hemophilus influenzae*, *Pseudomonas aeruginosa*, *Proteus*, *Bacteroides*, *Staphylococcus epidermidis*, and others [7]. In previous studies ([6,8]; Mariani et al. 1996b; [7]), we have demonstrated the usefulness of these 16S rRNA gene primers for the detection of species from a range of different genera including some of the species mentioned above. In this study, we have further shown the ability of these same primers to document the presence of *Staphylococcus aureus* in extracts derived directly from animal bone biopsies, thereby expanding the potential application of this technology for tissues and fluids. With the second set of animals in this study, the species-specific PCR primers that target the unique, *Staphylococcus aureus* thermostable nuclease (*nuc*) gene were used. This approach allowed not only for the rapid diagnosis of bacterial infection, but also verified that *Staphylococcus aureus* was the infectious agent, a situation that simulates the common conditions of chronic osteomyelitis in humans. In an analogous manner, the use of species-specific primers targeting other pathogens, such as those mentioned above for which extensive sequence information is available, would expand the diagnostic capability of the PCR-based molecular technology, i.e., the capability of speciation.

Limitations of this study include the small number of subjects, as this is an animal study. There was also discordance in findings for animals with low levels of infection. For the animals inoculated with 1 × 10^6^ CFU of bacteria, there was complete correlation between radiographic, microbiological and molecular testing. However, for animals receiving 1 × 10^4^ CFU inoculations, discordance between the different assays was observed. Whereas radiographic and molecular testing successfully identified all infected animals in the set, microbiological testing was negative in 2 of 7 animals. In one of the rabbits, #13, no bacterial outgrowth was detected in any of the four biopsy specimens from the osteotomy site or from the adjacent metaphysis; on the other hand, the PCR test yielded at least one positive test from each site. For the animals that received the lower titer inocula, i.e., 1 × 10^3^ or 1 × 10^2^ CFU, and developed early stage disease, microbiological testing had a particularly poor success rate. Only 1 of 4 animals gave a positive bacteriological culture test; in fact, of the 16 specimens obtained with the different biopsy methods, only 1 test was positive. The molecular methodology gave 8 positive tests from the same set of biopsies, including from the 1 specimen that was determined to be positive microbiologically. Of all the experimental animals, there was only one case (rabbit #10) in which a biopsy specimen from an infected animal gave a positive microbiological culture, and a negative PCR test; however, the three remaining biopsies from the same animal were positive by PCR, indicating unequivocally that this animal was indeed infected. Radiographic assessment was at best ambiguous in these animals with mild, low-level infection. Additionally, in the laboratory setting, sample processing is available immediately after biopsying tissue samples. In the clinical setting, it is recommended to biopsy all bone suspicious for osteomyelitis (cortical bone, cancellous bone, and medullary canal) and send it off for immediate processing on the same day as sampling. If immediate processing is not possible, we recommend that tissue samples be stored at 4°C to minimize bacterial growth and proliferation.

## Conclusion

Taken together with our previous studies ([6,8]; Mariani et al. 1996b; [7,13,14]), the findings reported here suggest the following practical approach to applying the PCR amplification technology for the diagnosis of infectious diseases. PCR and qPCR based testing with universal primers is to be used as the first screen of clinical specimens to identify those samples containing bacterial DNA, regardless of species, to alert the clinician that infection is highly probable in those patients testing positive. The limitation of PCR is that speciation is not possible and can limit antibiotic susceptibility data. A second round of amplification may then be performed on the positive specimens identified in the first screen, using an adequate number of multiplex amplification reactions employing multiple, species-specific primer sets, each corresponding to suspected pathogens as a means of species identification. Finally, as suggested by our recent refinements of the PCR test, the viable bacteria load may be assessed using a reverse-transcription based PCR protocol that targets viability-sensitive bacterial mRNAs [14] and 16S rRNA [13].

### Ethical board review

This study was conducted with Institutional Animal Care and Use Committee approval (Thomas Jefferson University IACUC) (Protocol 234 J).

## Abbreviations

CFU: Colony forming units
DNA: Deoxyribonucleic acid
PCR: Polymerase chain reaction
rRNA: Ribosomal ribonucleic acid
qPCR: Real time-quantitative PCR
